# Amino acid restriction sensitizes lung cancer cells to ferroptosis via GCN2-dependent activation of the integrated stress response

**DOI:** 10.1016/j.redox.2025.103988

**Published:** 2025-12-24

**Authors:** Viktor Antonsson Garellick, Nadia Gul, Parvin Horrieh, Dyar Mustafa, Angana A.H. Patel, Martin Dankis, Samantha W. Alvarez, Johanna Berndtson, Saeed Mahdavi, Maria Schwarz, Andreas Persson, Fikret Zahirovic, Clotilde Wiel, Volkan I. Sayin, Per Lindahl

**Affiliations:** a Sahlgrenska Center for Cancer Research, University of Gothenburg, Gothenburg, 405 30, Sweden; b Department of Molecular and Clinical Medicine, Institute of Medicine, University of Gothenburg, Gothenburg, 405 30, Sweden; c Department of Medical Biochemistry and Cell Biology, Institute of Biomedicine, University of Gothenburg, Gothenburg, 405 30, Sweden; d Department of Surgery, Institute of Clinical Sciences, University of Gothenburg, Gothenburg, 405 30, Sweden; e The Wallenberg Centre for Molecular and Translational Medicine, at Sahlgrenska Academy, University of Gothenburg, Gothenburg, 405 30, Sweden; f Department of Nutritional Physiology, Institute of Nutritional Sciences, Friedrich Schiller University Jena, Dornburger Str. 24, Jena, 07743, Germany

**Keywords:** Lung cancer, Amino acids, Glutathione, Ferroptosis, Integrated stress response, Mitochondrial respiration

## Abstract

Lung cancer cells are vulnerable to iron-dependent oxidation of phospholipids leading to ferroptosis, a process countered by glutathione peroxidase-4 that converts lipid hydroperoxides to lipid alcohols using glutathione as reducing agent. Since ferroptosis-inducing agents are in clinical development, identifying modifiers of ferroptosis susceptibility is warranted. Here, we investigate the impact of amino acids on susceptibility to buthionine sulfoximine (BSO), a glutamate-cysteine ligase inhibitor that blocks biosynthesis of glutathione. We found that reduced amounts of amino acids other than cysteine increased the sensitivity to BSO and other ferroptosis-inducing agents, in a panel of mouse and human lung cancer cells, without affecting glutathione production. Activation of the amino acid sensor protein GCN2 and the integrated stress response lowered the threshold for lipid peroxidation by promoting ATF4-dependent mitochondrial respiration and reactive oxygen species leakage from the electron transport chain under glutathione depletion. The finding provides new insights into lung cancer metabolism and raises the possibility of using amino acid restricted diets in combination with ferroptosis-inducing agents as cancer therapies.

## 1 Introduction

The cellular redox environment is firmly controlled and deviations from homeostasis may cause oxidative stress, a state where the amount of reactive oxygen species (ROS) exceeds the antioxidant capacity. Oxidative stress has emerged as a limiting factor for cancer progression and metastasis. Lung cancer cells are particularly sensitive to oxidative stress, as evidenced by the increased lung cancer risk observed in clinical trials of dietary antioxidants [1,2], frequent somatic mutations in nuclear factor erythroid 2-related factor 2 (NFE2L2, NRF2) or kelch-like ECH-associated protein 1 (KEAP1) that enhance production of endogenous antioxidants [[3], [4], [5]], and supporting mechanistic studies in mice [6,7]. Prooxidant cancer therapies that target endogenous antioxidants may therefore be effective against lung cancer.

Glutathione is the most abundant intracellular antioxidant and a major determinant of the cellular redox environment [8]. One important role of glutathione is to maintain the integrity of phospholipid membranes. Glutathione peroxidase-4 (GPX4) plays a key role in this process by converting harmful lipid hydroperoxides to benign lipid alcohols, using glutathione as reducing agent [9]. Depletion of glutathione leads to ferroptosis, a regulated form of cell death that is triggered by iron-dependent lipid peroxidation [10]. Ferroptosis has emerged as a central non-apoptotic cell death mechanism in cancer, and ferroptosis-inducing agents are considered for clinical development [11]. Identifying factors that govern susceptibility to ferroptosis-inducing agents could lead to improved therapeutic options.

Cultured human cancer cells are surprisingly resilient to glutathione depletion [[12], [13], [14]]. This can be explained by compensatory upregulation of the thioredoxin system [[15], [16], [17], [18]], but other factors may contribute. Tumor angiogenesis produces abnormal blood vessels leading to hypoxia and nutrient stress in tumors. Results obtained with culture media designed to optimize cell growth, such as the aforementioned studies, may underestimate the impact of nutrient stress on ferroptosis.

Ferroptosis susceptibility is modified by lipid metabolism, iron homeostasis, and amino acid metabolism, the latter mainly by influencing levels of glutathione [11]. The tripeptide glutathione is synthesized from glutamate, cysteine, and glycine in reactions that are limited by cysteine availability, and is hence vulnerable to perturbations affecting cysteine metabolism [19,20]. However, amino acids are major modifiers of drug resistance that intersect with drug mechanisms in abundant and variable fashions [21,22]. Whether amino acids modify ferroptosis susceptibility in additional ways beyond glutathione synthesis warrants further investigation.

Here, we investigate the impact of levels of amino acids on sensitivity to BSO, an inhibitor of glutamate-cysteine ligase catalytic subunit (GCLC; EC 6.3.2.2), the rate limiting enzyme in *de novo* biosynthesis of glutathione [23,24].

## 2 Materials and methods

### 2.1 Cell culture

The cell lines A549, NCIH838 (H838), NCIH1299 (H1299), NCIH23 (H23), NCIH460 (H460), SKNEB2, and SHSY5Y were obtained from ATCC. Cas9-expressing A549 cells were obtained from GeneCopoeia (SL-504; GeneCopoeia, Inc., Rockville, MD) and the mouse KP cell line was obtained from V. Sayin and is described [25].

A549, H838, H1299, H23, and H460 cells were maintained in Ham's F12 mixture (SH30026.01; Hyclone) or RPMI1640 (SH30027.01; Hyclone), supplemented with 10 % FBS and 1 % penicillin/streptomycin (SV30010; Hyclone). SKNEB2 and SHSY5Y were maintained in F12:MEM (1:1) supplemented with 10 % FBS and 1 % penicillin/streptomycin. KP cells were maintained in DMEM with 10 % FBS and 0.1 % gentamicin (15710049; Gibco). Experiments on KP cells were carried out in F12 or RPMI supplemented with 10 % FBS and 0.1 % gentamicin.

Custom-made F12AA and RPMIAA (Genaxxon bioscience, Germany; see Table 1 for medium formulation) were supplemented with 35.15 mg/L final concentration of l-cysteine-hydrochloride-monohydrate (C6852; Sigma Aldrich), 146 mg/L final concentration of l-glutamine (25030-081; Gibco), 1 % penicillin/streptomycin, and 10 % FBS. Cells were switched to experimental conditions one passage prior to the experiment unless otherwise specified in the text. Cells were kept at 37 °C and 5 % CO_2_^.^

**Table 1:** Cell culture media composition Table.

Component (mg/L)	F12	F12AA	RPMI	RPMIAA
**Amino acids**				
l-Alanine	8.91			8.91
l-Arginine-HCl	210.7	200	200	210.7
l-Asparagine-H2O	15.01	50	50	15.01
l-Aspartic acid	13.3	20	20	13.3
l-Cystine-2HCl			65.15	
l-Cysteine-HCl-H2O	35.12	35.12		35.12
l-Glutamic acid	14.7	20	20	14.7
l-Glutamine	146.2	146.2	300	146.2
Glycine	7.51	10	10	7.51
l-Histidine-HCl-H2O	20.96	15	15	20.96
l-Isoleucine	3.94	50	50	3.94
l-Leucine	13.12	50	50	13.12
l-Lysine-HCl	36.54	40	40	36.54
l-Methionine	4.48	15	15	4.48
l-Phenylalanine	4.96	15	15	4.96
l-Proline	34.53	20	20	34.53
l-Serine	10.51	30	30	10.51
l-Threonine	11.91	20	20	11.91
L-Tryptophan	2.04	5	5	2.04
l-Tyrosine-2Na–2H2O	7.78	28.83	28.83	7.78
l-Valine	11.71	20	20	11.71
**Inorganic salts**				
CaCl2 (anhydrous)	33.22	33.22		
CuSO4 x 5H2O	0.003	0.003		
Ca(NO3)2 × 4H2O			100	100
FeSO4 x 7H2O	0.834	0.834		
KCl	223.60	223.60	400	400
MgCl2 x 6H2O	122.00	122.00		
MgSO4			48.84	48.84
NaCl	7599.00	7599.00	6000	6000
Na2HPO4	142.04	142.04	800.49	800.49
ZnSO4 x 7H2O	0.86	0.86		
NaHCO3	1176.00	1176.00	2000	2000
**Other components**				
Glutathione (reduced)			1	1
Hypoxanthine	4.08	4.08		
Linoleic acid	0.084	0.084		
DL-α-Lipoic acid	0.21	0.21		
Putrescine x 2HCl	0.16	0.16		
Phenol red	1.20	1.20	5.30	5.30
Sodium pyruvate	110.00	110.00		
Thymidine	0.73	0.73		
d(+)-Glucose (anhydrous)	1801.60	1801.60	2000	2000
**Vitamins**				
p-Aminobenzoic acid			1	1
D(+)-Biotin	0.007	0.007	0.20	0.20
D-Ca-pantothenate	0.24	0.24	0.25	0.25
Choline chloride	13.96	13.96	3	3
Folic acid	1.32	1.32	1	1
myo-Inositol	18.02	18.02	35	35
Nicotinamide	0.037	0.037	1	1
Pyridoxol x HCl	0.062	0.062	1	1
Riboflavin	0.038	0.038	0.20	0.20
Thiamine x HCl	0.34	0.34	1	1
Vitamin B12	1.36	1.36	0.005	0.005

BSO sensitivity in F12 medium was influenced by the batch of FBS used (Fig. S5). The vast majority of experiments were performed with one FBS batch (Cat No. SV300160.03, Lot No. RB35958; Hyclone). A subset of experiments shown in (Fig. 7E, F, H, I, J, K, L, N) and (Fig. S4C and D) and (Fig. S9F and G) were conducted with another FBS batch (Cat No. SV30160.03, Lot No. RB35957; Hyclone).

### 2.2 Cell growth

1.5 × 10^5^ cells/well were seeded in a 6-well plate in F12 or RPMI medium. On the first passage, after three days, the cells were counted using a trypan blue exclusion assay. 1.5 × 10^5^ cells/well were then re-seeded and counted again for two more passages.

### 2.3 7-AAD staining

1.5 × 10^5^ cells/well were seeded overnight in a 6-well plate. The next day, cells were incubated in 5 μM etoposide overnight to induce apoptosis, after which the cells were washed in cold PBS and incubated with 1 ng/μL 7-AAD (A1310; Thermo Fisher) for 30 min on ice. The 7AAD signal was quantified with a flow cytometer (BD Accuri C6) at (λex488 nm) using the FL3 detector. The cut-off value for 7-AAD positive cells were based on the signal distribution in etoposide-treated cells versus untreated negative controls.

### 2.4 Cell viability

For crystal violet viability assays, 2 × 10^4^ cells/well were seeded overnight in 12-well plates, incubated with drugs dissolved in fresh media for 72 h, and stained with 0,025 % (v/v) of crystal violet staining solution (V5265; Sigma Aldrich) supplemented with 1 % (v/v) formaldehyde, 1 % (v/v) methanol and diluted in PBS, for 20 min. After staining, the plates were washed with tap water and air dried for a minimum of 24 h. Once dried, the staining solution was re-dissolved with 10 % acetic acid and light absorbance was measured at 595 nm using a SpectraMax MiniMax spectrophotometer. Absorbance was subtracted for background and then normalized to untreated control.

CellTiter-Glo® (G7570; Promega) assays were performed according to the manufacturer's instruction. Briefly, 2 × 10^3^ cells/well were seeded overnight in white opaque-bottom 96-well plates, incubated with drugs in fresh medium for 72 h, and stained with CellTiter-Glo® reagent for 10 min. Luminescence was measured with a BioTek Synergy HTX plate reader. Luminescence was normalized to untreated control.

### 2.5 Drug treatments

Drugs used were auranofin (A6733; Sigma-Aldrich), α-tocopherol (T3251; Sigma-Aldrich), bafilomycin A1 (SML1661; Sigma-Aldrich), certolizumab pegol (HYP9953; MedChemExpress), chloroquine (C6628; Sigma-Aldrich), CU-CPT4A (HY-108473; MedChemExpress), deferoxamine (D9533; Sigma-Aldrich), erastin (e7781; Sigma Aldrich), FCCP (Carbonyl cyanide 4-(trifluoromethoxy)phenylhydrazone) (HY-100410; MedChemExpress), ferrostatin-1 (SML0583; Sigma-Aldrich), l-buthionine-sulfoximine (b2515; Sigma-Aldrich), mito-TEMPO (HY–W001187; MedChemExpress), necrostatin-1 (N9037; Sigma-Aldrich), liproxstatin-1 (SML1414; Sigma-Aldrich), oligomycin (HY–N6782; MedChemExpress), puromycin dihydrochloride (A1113803; Gibco), rotenone (HY–B1756; MedChemExpress), RSL3 (SML2234; Sigma-Aldrich), resatorvid (HY-11109; MedChemExpress), and Z-VAD-FMK (V116; Sigma-Aldrich).

### 2.6 Western blotting

Semi-confluent cells were lysed with 8 M Urea buffer or RIPA buffer supplemented with Pierce protease and phosphatase inhibitor cocktail (A32959; Thermo Fisher Scientific), centrifuged at 20000 rcf for 10 min at 4 °C, mixed with LDS loading buffer supplemented with 10 mM DTT or 2.5 % β-mercaptoethanol, and incubated at 70 °C for 10 min. Proteins were separated on SDS-PAGE gels at 200 V and transferred onto nitrocellulose membranes using semi-dry transfer. Membranes were blocked with EveryBlot blocking buffer (12010020; Bio-Rad) for 5 min, or with 5 % milk in TBS-T buffer for 60 min. Primary antibody incubation was done overnight in blocking buffer at 4 °C and secondary antibody incubation at room temperature for 1 h. Membranes were incubated with Clarity™ Western ECL substrate (1705060; Bio-Rad) for 5 min. Chemiluminescent imaging was done using a GE Amersham Imaging device. Band intensities were quantified with the ImageJ software. Primary antibodies used were 4E-BP1 (9452; Cell signaling technology (CST)), ATF4 (11815; CST), α-Tubulin (T6199; Sigma-Aldrich), CHAC1 (15207-1-AP; Proteintech), CHOP (15204-1; Proteintech), eIF2α (5324; CST), Ferritin (ab75973-1001; Abcam), GADD34 (10449-1-AP; Proteintech), GAPDH (5174; CST), GCN2 (3302; CST), GCLC (ab41463; Abcam), LC3B (NB100–2220SS; Novusbio), p-4E-BP1 (2855; CST), p-eIF2α Ser 51 (3398; CST), p-GCN2 (ab75836; Abcam), p-S6 (2211; CST), S6 (2217; CST), TfrC (13–6800; Invitrogen), p-PKR (ab32036; Abcam), p-PERK (ab192591; Abcam), PERK (3192; CST), PKR (sc-6282; SCBT), and Hsp90 (4877; CST). Secondary antibody used was peroxidase-conjugated AffiniPure Goat Anti-Rabbit IgG (H + L) (111-035-003; Jackson Immunoresearch) and peroxidase-conjugated AffiniPure Goat Anti-Mouse IgG (H + L) (115-035-003; Jackson Immunoresearch).

### 2.7 qPCR

Total RNA was isolated from A549 cells transfected with siRNA 24 h post-transfection, using RNeasy mini kit (Qiagen) according to the manufacturer's protocol. The purity and concentration of the extracted RNA were assessed using a NanoDrop spectrophotometer. cDNA was synthesized from 300 ng of total RNA using the iScript™ Reverse Transcription Supermix for RT-qPCR (Biorad) according to the manufacturer's protocol. The reverse transcription reaction was carried out in a thermal cycler at 25 °C for 5 min, followed by 46 °C for 20 min, and then 95 °C for 1 min to inactivate the enzyme. qPCR reactions were performed in a 20 μL volume containing 2 μL of cDNA, 10 μL of PowerUp™ SYBR™ Green Master Mix (Thermo Fisher), 300 nM of each primer, and nuclease-free water to a final volume of 20 μL. Primer pairs against ASNS, SLC7A11, CHAC1 and DDIT3/CHOP were purchased from Sigma-Aldrich (KiCqStart™ Primers ID: ASNS ID H_ASNS_1, SLC7A11 ID H_SLC7A11_1, DDIT3/CHOP H_DDIT3_1, CHAC1 H_CHAC1_1). Primers pairs against GAPDH was purchased from IDT technologies (Forward primer 5′-CGC TCT CTG CTC CTC CTG TT-3′ and Reverse primer 5′- CCA TGG TGT CTG AGC GAT GT-3′). The qPCR was carried out in an Applied Biosystems 7900 Real-Time PCR System with cycling conditions as per manufacturer's recommendations. Gene expression levels were normalized to GAPDH and analyzed using the 2^-ΔΔCt method. No-template controls were included in each run, and melting curve analysis was performed to ensure specificity of the PCR products.

### 2.8 Generation of GCLC knockout cells

GCLC knockout cells were generated with CRISPR-Cas9, as in Ref. [26]. Briefly, guide-RNA (gRNA) spacer sequences targeting constituently expressed exons in GCLC were designed using the Benchling tool (https://www.benchling.com). Double-stranded DNA oligonucleotides (20 nt) were cloned into the BamH1 site of the Lentiguide puro vector, and co-transfected with pCMV-R8.2 and pCMV-VSV-G into HEK293T cells to produce gRNA coding lentivirus. Cas9-expressing A549 cells were infected with virus and cultured in DMEM supplemented with puromycin for 7 days to produce knockout batch clones (verified with western blotting at day 7; Fig. S1E) that were used in experiments. The cells were switched to F12 after day 7 and evaluated for viability at day 13. Lentiguide puro was a kind gift from Feng Zhang (Addgene plasmid #52963; http://n2t.net/addgene: 52963; RRID:Addgene_52963) [27]. Oligonucleotide sequences used for the creation of gRNA constructs were: GCLC gRNA#1: forward oligo CACCGCATACTCACCTGAAGCGA, reverse oligo AAACTCGCTTCAGGTGAGTATGC; GCLC gRNA#2: forward oligo CACCGTAGATGTGCAGGAACTGG, reverse oligo AAACCCAGTTCCTGCACATCTAC; GCLC gRNA#3: forward oligo CACCGAAATATCCGACATAGGAG, reverse oligo AAACCTCCTATGTCGGATATTTC; control gRNA: forward oligo CACCGGAGGCTAAGCGTCGCAA, reverse oligo AAACTTGCGACGCTTAGCCTCC.

### 2.9 CM-H2DCFDA ROS measurements

For estimation of general ROS levels, A549 cells were seeded at a density of 1 × 10^5^ cells per well in a 6-well plate containing RPMI or F12 media and allowed to attach overnight. The cells were then treated with or without 100 μM BSO for 24 h. As positive controls, cells were treated for 10 min with 25 μM Menadione. Following treatments, the cells were harvested using trypsin and subsequently incubated for 30 min at 37 °C with 5 μM CM-H2DCFDA solution (C6827; Thermo Fisher Scientific). The cells were then washed three times with PBS, resuspended in 200 μL of PBS, and flow cytometry data was collected and analyzed using a BD Accuri™ C6 Plus Flow Cytometer.

### 2.10 Lipid peroxidation analysis by flow cytometry

Lipid peroxidation was estimated using the lipid soluble redox-sensitive BODPIPY-C11™ sensor (D3861, Thermo Fisher) as in Ref. [28]. A549 or H838 cells were plated in 6-well plates at 1 × 10^5^ and 2 × 10^5^ cells/well, respectively, in the presence of 100 μM BSO or vehicle. 24-hours post plating, BODIPY-C11 was added to the culture medium to a final concentration of 1.5 μM, after which the cells were stained for 20 min at 37 °C. The amount of oxidized BODIPY-C11 was quantified with a flow cytometer (BD Accuri C6) at (λex488nm) using the FL1 detector. To define BODIPY-C11 positive cells, a cut-off value was calculated based on the signal distribution in positive (cells treated with 5 μM erastin for 6 h) and negative (untreated cells) controls.

### 2.11 Glutathione determination

Glutathione (reduced) concentrations were determined using the GSH-Glo™ Glutathione assay (V6911; Promega corporation) according to the manufacturer's instruction. 2 × 10^3^ cells/well were seeded overnight in white opaque-bottomed 96-well plates. The next day, cells were incubated with BSO (the concentrations are indicated in the graphs) in fresh medium for 24 h, after which GSH-Glo and luciferin detection reagents were added. Luminescence was measured using a BioTek Synergy HTX plate reader.

### 2.12 Gas chromatography-mass spectrometry (GC/MS) analysis of amino acids

For the amino acid uptake assay, 1 × 10^5^ cells were seeded in 2 mL of their respective cell culture medium with 10 % FBS in 6-well plates for two days before harvest. For relative determination of intracellular amino acid levels, 1 × 10^5^ cells were seeded in 2 mL of RPMI and left to attach overnight. The next day, spent cell medium was replaced with fresh F12, F12AA or RPMI medium. Lysates were collected at 1, 6, 24 and 48 h post medium renewal by scraping the cells in 200 μl of 80 % (v/v) ice cold methanol containing 1 μg/mL norvaline (Sigma-Aldrich). All Samples were then vortexed for 10 min at 4 °C and then centrifuged at 20000 rcf for 10 min. The supernatant was transferred to fresh tubes and solvents were evaporated in a speed vac for 2 h. Dried metabolite extracts were subsequently derivatized with 20 μL O-methoxyamine-hydrochloride (MOX) reagent (Sigma-Aldrich) in pyridine (Sigma-Aldrich) at a concentration of 20 mg/mL for 60 min at 37 °C followed by 20 μL of N-tert-butyldimethylsilyl-N-methyltrifluoracetamide with 1 % tert-butyldimethylchlorosilane (TBDMS, Sigma-Aldrich) for 60 min at 37 °C. After derivatization, samples were analyzed by GC-MS using an DB-35 ms column (Agilent Technologies) in an Agilent Intuvo gas chromatograph coupled to an Agilent 5997B mass spectrometer. Helium was used as the carrier gas at a flow rate of 1.2 mL/min. 1 μl of sample was injected in split mode (split 1:1) at 270 °C. After injection the GC oven was held at 100 °C for 1 min and then increased to 300 °C at 3.5 °C/min. The oven was then ramped to 320 °C at 20 °C/min and held for 5 min at 320 °C. The MS system operated under electron impact ionization at 70 eV and the MS source and quadrupole were held at 230 °C and 150 °C respectively. The detector was used in scanning mode, and the scanned ion range was 10–650 *m*/*z*. Mass isotopomer distributions were determined by integrating the appropriate ion fragments for each metabolite [29] using MATLAB (Mathworks) and an algorithm adapted from Fernandez and colleagues [30] that corrects for natural abundance. For the uptake assay, total metabolite pool sizes were normalized to cell counts for each condition separately. For intracellular amino acid determination, total metabolite pool sizes were normalized to total protein content determined by Pierce BCA assay for each condition separately.

### 2.13 Overexpression of CHOP and ATF4

To achieve overexpression of CHOP and ATF4, the plasmids TFORF0623 and TFORF3036 were used respectively. Both plasmids were obtained from Addgene (plasmid #142666 and plasmid #144512, respectively) as gifts from Feng Zhang. Lentiviral particles were produced (as described above) from these plasmids and used to transduce A549 cells. Following transduction, the culture medium was replaced after 24 h and cells were selected in puromycin for 3 days to isolate successfully transduced populations. The selected cells were subsequently used in downstream experiments to assess the effects of CHOP and ATF4 overexpression.

### 2.14 siRNA transfection

2 × 10^5^ or 2 × 10^3^ cells/well were seeded overnight in 6- or 96-well plates, respectively, and then transfected with 25 or 1 pmol siRNA duplexes (or a universal negative control) using Lipofectamine ™ RNAiMax transfection reagent (13778100; Thermo Fisher Scientific), according to the manufacturer's instruction. 24–48 h after transfection, the cells were used in experiments or harvested for downstream analyses. siRNAs used were SASI_HS01_00097889 (GCN2), SASI_HS01_00097888 (GCN2), SASI_HS01_00097890 (GCN2), SASI_HS02_00332313 (ATF4), SASI_HS02_00332314 (ATF4), SASI_HS01_00175197 (ATF4), SASI_HS01_00153013 (CHOP), SASI_HS02_00336880 (CHOP), SASI_HS01_00153015 (CHOP), SASI_Hs01_00237406 (GADD34), SASI_Hs02_00345455 (GADD34), SASI_Hs02_00345456 (GADD34), SASI_Hs01_00146246 (CHAC1), SASI_Hs01_00146247 (CHAC1), and SASI_Hs01_00146249 (CHAC1) (Sigma-Aldrich).

### 2.15 Mitochondrial respiration

Analyses of mitochondrial respiration were performed using the Seahorse XFe96/XFPro Analyzer (Agilent), which measures the extracellular acidification rate (ECAR) and oxygen consumption rate (OCR) of live cells. Mitochondrial stress was measured using the following compounds: 0.5 μM oligomycin, 1 μM FCCP, and 0.5 μM rotenone. Cells were maintained in their respective media for at least 48 h before analysis. The night before the assay, cells were seeded into Seahorse XFe96/XF Pro Cell Culture Microplates (1 × 10^4^ cells/well; Agilent, 103792-100) and cultured overnight in F12 or F12AA medium at 37 °C in a CO_2_ incubator. Forty-five minutes prior to the assay, the medium was replaced with freshly prepared phenol red-free RPMI medium (Agilent, 103576-100) supplemented with 1 mM glucose and 2 mM glutamine. The Seahorse Analyzer was pre-warmed to 37 °C before use. ATP production rates were calculated using the Excel macro provided by the manufacturer.

### 2.16 Live imaging of MitoSox, BODIPY-C11, and MitoPerOx

2 × 10^3^ cells/well were seeded in 96-well plates and allowed to adhere overnight in the appropriate culture medium. The following day, cells were incubated for 1 h with 0.1 μM MitoSox Red (HY-D1055; MedChemExpress), 1.5 μM BODIPY-C11, or 0.1 μM MitoPerOx (HY-125623; MedChemExpress), washed with PBS, and replenished with fresh medium supplemented with the indicated reagents or drugs. Plates were then placed in the IncuCyte live-cell imaging system (Model 2022B Rev2). Phase-contrast and fluorescence images were acquired at regular intervals (typically every 4–6 h) for the duration of the experiment and analyzed using the integrated IncuCyte software.

For ratiometric confocal microscopy, images were acquired from live cells on an LSM 980 (Zeiss) using a 63x/1.40 oil immersion plan-apochromatic objective lens kept at 37 °C. For combination of BODIPY-C11 and Mitotracker DeepRed (Invitrogen), a sequential acquisition was setup with the first track excitation at 561 nm and detection from 552 to 640 nm, imaging the oxidized BODIPY-C11, and the second track excitation at 488 and 639 nm and detection from 493 to 517 nm and 653–668 nm, imaging the reduced BODIPY-C11 and the Mitotracker DeepRed. For MitoPerOx, acquisition was set up with excitation at 488 nm and detection at 520 nm (oxidized state) and 590 nm (reduced state). 7–16 random fields per well were imaged using the ZEN software Sample Navigator for unbiased sampling.

### 2.17 Statistics

Statistical analyses were done with GraphPAD Prism 9 (GraphPad Software, San Diego, CA, USA). Difference between two groups was tested with unpaired student's t-test, difference between multiple groups and treatments was tested with multiple unpaired *t*-test, one-way ANOVA or two-way ANOVA using Dunnet's or Tukey's post-hoc test (main row effect), and difference in LC3-II levels and cell growth was tested with multiple unpaired t-tests. Autophagy flux was calculated using linear regression. IC50 values were calculated using non-linear regression analysis of inhibitor versus response: variable slope (four parameters). *P*-values <0.05 was considered significant. Data are presented as mean ± SEM.

## 3 Results

### 3.1 Nutritional factors influence the susceptibility to ferroptosis-inducing agents in cancer cells

**Fig. 1:**
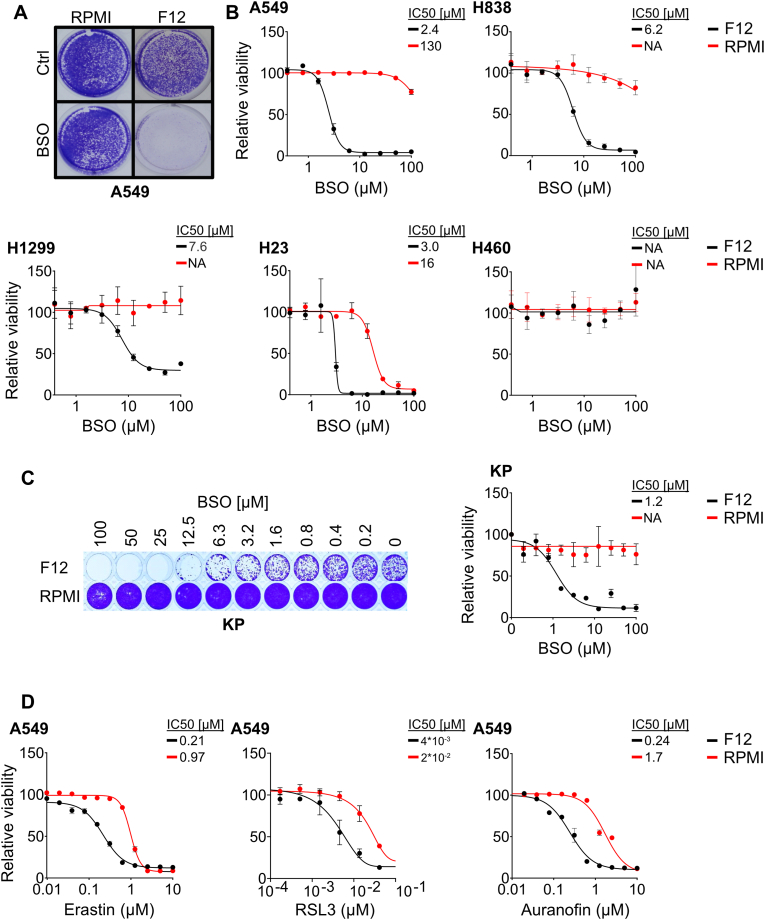
**Culture in F12 medium sensitizes lung cancer cells to BSO.** (**A**) Crystal violet staining of A549 cells that were cultured in RPMI or F12 medium, after treatment with 100 μM BSO or vehicle (Ctrl) for 72 h. (**B**) BSO dose response curves for A549, H838, H1299, H23, and H460 cells cultured in RPMI or F12 medium for 72 h. (**C**) Crystal violet staining and quantification of mouse KP cells that were cultured in RPMI or F12 medium in the presence of BSO at the indicated concentrations for 72 h. (**D**) Dose response curves for A549 cells cultured in RPMI or F12 medium and treated with erastin, RSL3, or auranofin for 72 h. Dose response curves were normalized against the mean of the untreated samples for each condition. n = 3 replicates for all datapoints, error bars show SEM.

To investigate the impact of nutrient factors on susceptibility to ferroptosis-inducing agents, we established cultures of the human lung cancer cell line A549 in Ham's F12 nutrient mixture (F12) or RPMI, two culture media used in cancer research that differ in amino acid content. Culture in F12 medium retarded cell growth slightly compared to culture in RPMI medium (Fig. S1A) but had no effect on cell death or cell morphology (Fig. S1B and C). To assess the susceptibility to ferroptosis-inducing agents, we established dose response curves for BSO. While RPMI-cultured cells were strikingly resistant to BSO, F12-cultured cells died at low micromolar concentrations (Fig. 1A and B). Similar results were obtained for H838, H1299, and H23 lung cancer cells (Fig. 1B) indicating that nutrient factors have significant impact on BSO sensitivity.

Culture in F12 medium increased BSO sensitivity in mouse KP lung cancer cells (Fig. 1C) and human SKNBE-2 neuroblastoma cells compared to RPMI (Fig. S1D), showing that the mechanism is evolutionarily conserved and not restricted to lung cancer cells. The culture media had no impact on BSO sensitivity in human H460 lung cancer cells or SH-SY5Y neuroblastoma cells (Fig. 1B and Fig. S1D), indicating that intrinsic factors such as genetic makeup, epigenetic history, or cell-of-origin play a role.

To verify that BSO-lethality is caused by GCLC-inhibition, A549 cells were infected with lentivirus expressing sgRNAs targeting GCLC, or a non-targeting control. Knockout of GCLC was lethal in F12-cultured cells, consistent with the pharmacological data (Fig. S1E and F).

To test whether F12 increases the sensitivity to other ferroptosis-inducing agents, we established dose response curves for erastin, RSL3 and auranofin in F12 or RPMI-cultured A549 cells. The IC50 values of the drugs were consistently lower in cells that were cultured in F12 compared to RPMI (Fig. 1D). Thus, culture in F12 medium increases the susceptibility to several ferroptosis-inducing agents.

### 3.2 Culture in F12 medium lowers the threshold for iron-dependent lipid peroxidation

**Fig. 2:**
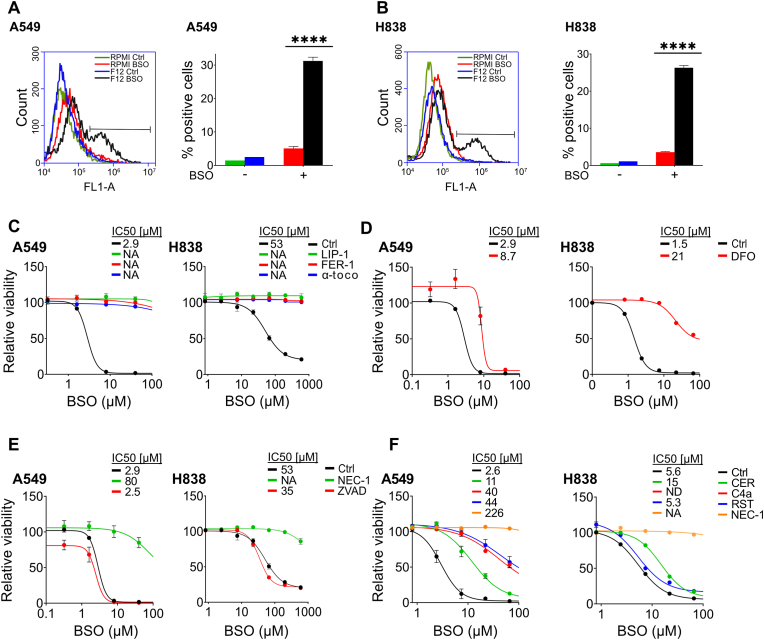
**F12 medium sensitizes lung cancer cells to iron-dependent lipid peroxidation**. (**A, B**) Quantification by flow cytometry of oxidized BODIPY-C11 fluorescence in A549 (A) or H838 (B) cells that were cultured in RPMI or F12 medium and treated with 100 μM BSO or vehicle for 24 h. (**C**) Dose response curves for F12-cultured A549 and H838 cells treated with BSO in combination with 5 μM liproxstatin-1 (LIP-1), 5 μM ferrostatin-1 (FER-1) or 50 μM α-tocopherol (α-toco) for 72 h. (**D**) Dose response curves for F12-cultured A549 or H838 cells treated with BSO in combination with 5 μM deferoxamine (DFO) for A549 cells and 9 μM for H838 cells for 72 h. (**E**) Dose response curves for F12-cultured A549 or H838 cells treated with BSO in combination with 5 μM necrostatin-1 (NEC-1) or 10 μM ZVAD-FMK (ZVAD) for 72 h. (**F**) Dose response curves for F12-cultured A549 or H838 cells treated with BSO in combination with 4 μg/mL certolizumab (CER), 15 μM CU-CPT4a (C4a), 3 μM resatorvid (RST), or 50 μM necrostatin-1 (NEC-1) for 72 h (ND, not done). Dose response curves were normalized against the mean of the untreated samples for each condition. n = 3 replicates for all datapoints, error bars show SEM. ∗∗∗∗P < 0.0001.

Since glutathione is required for reduction of lipid hydroperoxides to lipid alcohols by GPX4, depletion of glutathione may sensitize cells to lipid peroxidation [9,29]. Indeed, BSO treatment (100 μM, 24 h) dramatically increased fluorescence emission of the lipid peroxidation sensor BODIPY-C11 in F12-cultured cells, but only marginally in cells that were cultured in RPMI (Fig. 2A and B). There was no difference in general ROS levels between BSO-treated F12 and RPMI-cultured cells, as indicated by the CM-H2DCFDA sensor (Fig. S2). Moreover, F12-cultured cells were rescued from lethal BSO concentrations by the lipid peroxide scavengers liproxstatin-1, ferrostatin-1, or α-tocopherol (Fig. 2C). Since lipid peroxidation is catalyzed by ferrous iron in Fenton-like reactions [30], F12-cultured cells were BSO-treated in the presence of the iron chelator deferoxamine. Deferoxamine reduced BSO sensitivity in A549 and H838 cells (Fig. 2D).

Iron-dependent lipid peroxidation is indicative of ferroptosis but also causes other forms of regulated cell death. Cell death mechanisms are distinguished by their use of distinct effector proteins: caspases in apoptosis and pyroptosis, receptor-interacting serine/threonine protein kinase 1 (RIPK1) in necroptosis, and none of these in ferroptosis [31]. BSO-treated A549 or H838 cells that were cultured in F12 medium were rescued in the presence of the RIPK1 inhibitor necrostatin-1 but not in the presence of the pan-caspase inhibitor Z-VAD-FMK (Fig. 2E), suggesting that necroptosis might play a role.

RIPK1 operates downstream of tumor necrosis factor receptor-1 (TNFR1) or toll-like receptor (TLR) 3 or 4 in necroptosis [31]. In line with the necrostatin-1 results, BSO-treated A549 cells were rescued in the presence of the TNFR1 inhibitor certolizumab, the TLR3 inhibitor CU-CPT4a, or the TLR4 inhibitor resatorvid (Fig. 2F). BSO-treated H838 cells were similarly rescued in the presence of certolizumab (Fig. 2F), supporting the involvement of necroptosis. The cells were not rescued by resatorvid, and CU-CPT4a was toxic and therefore not tested, indicating some variation between the cell lines. Overall, the results suggest that F12-culture lowers the threshold for necroptosis in response to BSO-induced iron-dependent lipid peroxidation.

### 3.3 The sensitizing effect of F12 medium is caused by lower amino acid content

To identify which nutritional factors increase sensitivity to BSO in F12-cultured cells, we compared the composition of F12 and RPMI medium. F12 contains less amino acids compared to RPMI, but a wider variety of other components (Table 1). To test whether BSO sensitivity is governed by the amount of amino acids, we supplemented F12 medium with a standard mix of essential and non-essential amino acids to raise the total amino acid concentration to 3x that of RPMI, then generated BSO dose response curves. Addition of extra amino acids rescued A549 cells from BSO (Fig. S3A).

**Fig. 3:**
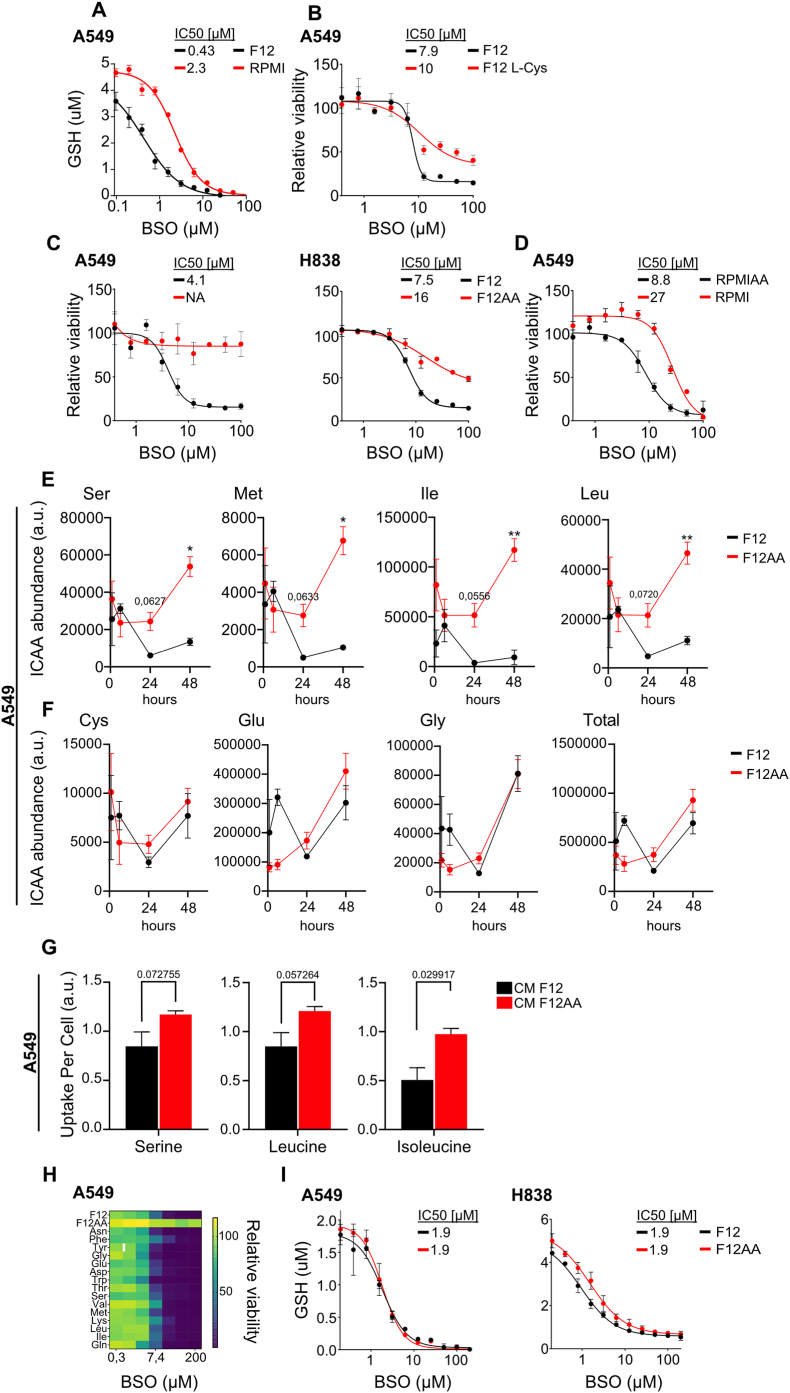
**The sensitizing effect of F12 medium is caused by lower amino acid content.** (**A**) Concentrations of reduced glutathione in lysates of A549 cells cultured in RPMI or F12 medium and treated with the indicated concentrations of BSO for 24 h. (**B**) BSO dose response curves for A549 cells cultured in F12 or F12 medium supplemented with 65 mg/L cystine (F12 L-Cys) for 72 h. (**C**) BSO dose response curves for A549 and H838 cells cultured in F12 or F12AA medium, the latter with amino acid concentrations matching those of RPMI (see Table 1), for 72 h. **(D)** BSO dose response curves for A549 cells cultured in RPMI or RPMIAA medium, the latter with amino acid concentrations matching those of F12 (see Table 1), for 72 h. (**E, F**) GC/MS data for intracellular levels of serine, methionine, isoleucine, and leucine (E) or cysteine, glutamate, and glycine (F) in A549 cells at 1, 6, 24, and 48 h after switching from RPMI medium to F12 or F12AA medium. The cells were maintained in RPMI and then passaged into fresh RPMI for 24 h before being switched to F12, F12AA, or fresh RPMI. **(G)** GC/MS data showing uptake of serine, leucine, and isoleucine in A549 cells that were cultured in F12 or F12AA medium for 48 h. **(H)** Heatmap showing BSO dose responses of A549 cells cultured in F12 medium supplemented with the indicated amino acids at final concentrations matching the ones in RPMI (see Table 1). **(I)** Concentrations of reduced glutathione in lysates of A549 and H838 cells cultured in F12 or F12AA medium and treated with the indicated concentrations of BSO for 24 h. Dose response curves were normalized against the mean of the untreated samples for each condition. n = 3 replicates for all datapoints, error bars show SEM. ∗∗P < 0.01, ∗P < 0.05.

The mass concentration of cystine (a dimer of oxidized cysteine) in RPMI exceeds the mass concentration of cysteine in F12. Since cystine is converted to cysteine in the cytoplasm and availability of cysteine limits glutathione production [24,32], we expected glutathione concentration and BSO tolerance to be higher in RPMI compared to F12-cultured cells. In agreement, concentrations of glutathione were higher in RPMI-cultured cells after titration with BSO (Fig. 3A), and addition of cystine increased the BSO tolerance of F12-cultured cells (Fig. 3B).

To test whether addition of amino acids other than cystine alters BSO sensitivity, we used a custom-made F12 medium (F12AA) with amino acid concentrations matching the ones in RPMI, with two modifications: We replaced cystine with cysteine at the same concentration as in F12 to minimize the impact on glutathione production. We also maintained the concentration of glutamine as in F12 since glutamine can affect ferroptosis susceptibility [33,34]. The formulations of the media used are shown in Table 1. The higher concentration of amino acids in F12AA conferred marked resistance to BSO in A549 cells, compared to F12-cultured cells (Fig. 3C). Similar results were obtained for H838 cells (Fig. 3C). Conversely, A549 cells cultured in a custom-made RPMI medium with amino acid concentrations as in F12 (RPMIAA) were more sensitive to BSO compared to RPMI-cultured cells (Fig. 3D).

To assess the media effect on amino acid levels, we determined the amounts of intracellular amino acids in RPMI-cultured A549 cells at different time points after feeding the cells with fresh medium, using gas chromatography-mass spectrometry (GC/MS). With fresh RPMI medium, the concentration of most amino acids increased with a peak at 24 h before returning toward baseline (Fig. S3B). In contrast, with F12 medium, amino acid concentrations decreased with a lowest point at 24 h, raising the possibility that intracellular amino acid depletion contributes to ferroptosis sensitivity (Fig. S3B). However, levels of intracellular amino acids differed between cells that were given RPMI and F12AA medium that have similar amino acid profiles (Fig. S3B), showing that media components other than amino acids have a major impact.

To pinpoint effects caused by the amino acid composition of the media, we compared intracellular amino acid concentrations in RPMI-cultured cells receiving fresh F12 or F12AA medium that differ only in amino acid composition. We hypothesized that critical amino acids (1) are depleted over time in F12-cultured cells and (2) are less abundant in F12 compared to F12AA-cultured cells. Four amino acids met these criteria: Levels of serine, methionine, leucine, and isoleucine decreased over time in F12-cultured cells and were lower in F12 compared to F12AA-cultured cells (Fig. 3E). The levels of cysteine, glycine and glutamate, which are precursors of glutathione, were not lower in F12-cultured cells compared to F12AA-cultured cells (Fig. 3F), suggesting that the substrates required for glutathione biosynthesis are unaffected. This result was expected, as the concentrations of these amino acids are similar in both media. Intracellular levels of all amino acids are shown in (Fig. S3B).

We then examined amino acid release and uptake by comparing amino acid levels in fresh and conditioned media (Fig. S4A). The highest uptake was recorded for serine, leucine, and isoleucine, indicating high consumption of these essential amino acids (Fig. 3G). The uptake was lower in F12 compared to F12AA-cultured cells, suggesting that limited availability contributes to the intracellular depletion of these amino acids. There was no difference in the uptake of methionine.

To test whether BSO sensitivity is governed by the media concentration of single amino acids, we supplemented F12 with one amino acid at a time to concentrations matching the ones in RPMI, and established BSO dose response curves for A549 cells. In contrast to extra cystine that increased BSO resistance (Fig. 3B), none of the other amino acids including serine, methionine, leucine, and isoleucine substantially affected BSO sensitivity (Fig. 3H and Fig. S4B). Similarly, supplementation of F12AA with alanine, the only amino acid uniquely present in F12, had no effect (Fig. S4C). Thus, BSO sensitivity is not governed by the concentration of single amino acids. To evaluate whether combinations of amino acids influence BSO sensitivity, F12 medium was supplemented with serine, methionine, leucine, and isoleucine, the four amino acids most depleted in cells cultured in F12, to match their concentrations in RPMI. This supplementation reduced BSO sensitivity in H838 cells but had no effect in A549 cells (Fig. S4D). These findings support a model in which the combined availability of multiple amino acids modulates cellular sensitivity to BSO.

Next, we investigated whether the difference in BSO sensitivity between F12 and F12AA-cultured cells reflected the availability of glutathione. There was no difference in glutathione concentration between F12 and F12AA-cultured A549 cells, and only a minor difference in H838 cells (Fig. 3I). Thus, F12AA-cultured cells remained healthy at BSO concentrations that virtually depleted the cells of glutathione. We conclude that a higher amino acid concentration confers resistance to BSO not by increasing glutathione availability, but by rendering cells independent of glutathione.

The BSO-sensitizing effect of F12 was not observed with all batches of FBS (Fig. S5A). With one batch, A549 cells proliferated for several passages in the presence of BSO before they died (Fig. S5B). The number of passages that passed before the cells died inversely correlated to the concentration of BSO. This result is reminiscent of a previous study in which BSO induced proteotoxic stress after prolonged treatment [12]. We did not investigate this phenomenon further.

### 3.4 Reduced amino acid levels activates the integrated stress response pathway

Amino acids are used in mRNA translation, as precursors in biosynthesis, as substrates for energy metabolism, and as signaling molecules. To assess whether amino acid signaling plays a role, we investigated the major amino acid sensing pathways: mammalian target of rapamycin complex 1 (mTORC1) – which is particularly sensitive to leucine and isoleucine – and general control nonderepressible-2 (GCN2).

**Fig. 4:**
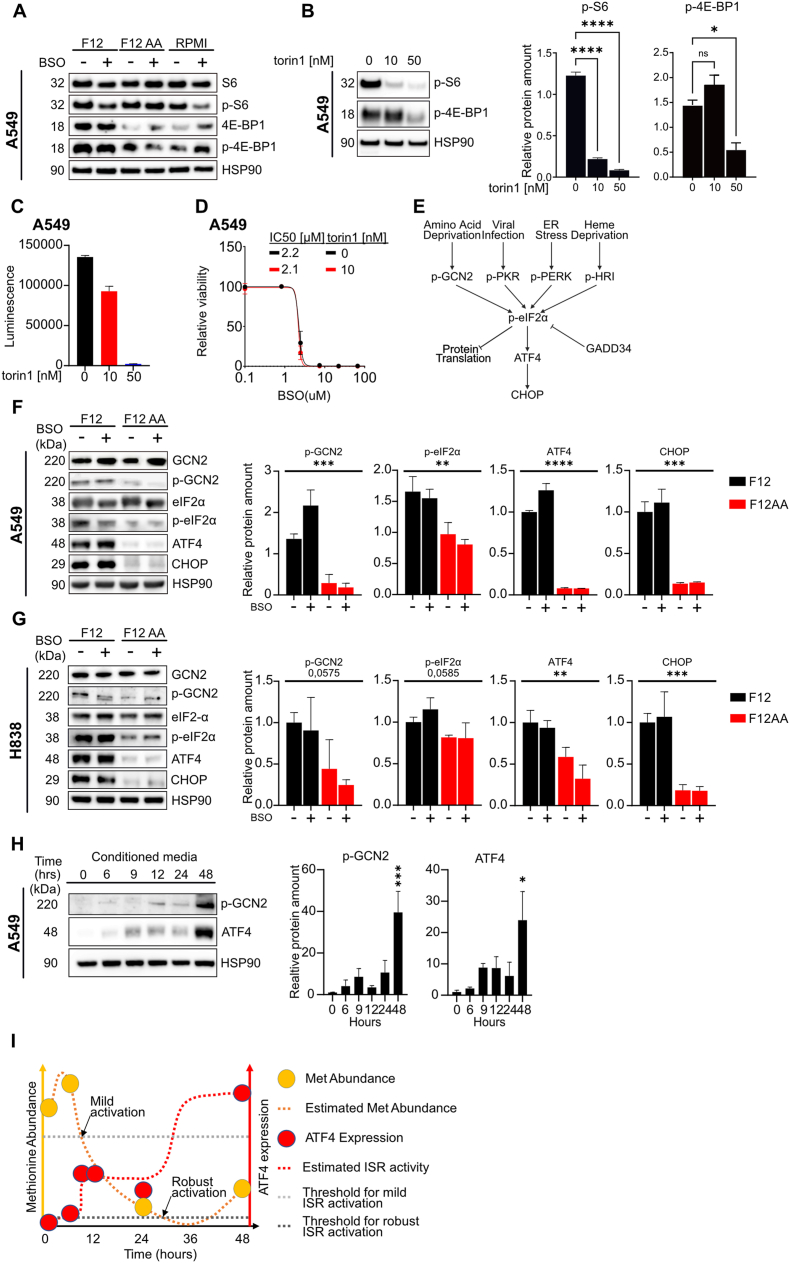
**The integrated stress response pathway is activated in F12-cultured cells**. (**A**) Western blotting of S6, p-S6, 4E-BP1, p-4E-BP1 in protein extracts of A549 cells cultured in F12, F12AA, or RPMI medium and treated with 100 μM BSO for 24 h. HSP90 was used as loading control. **(B)** Western blotting and quantification of p-S6 and p-4E-BP1 in protein extracts of A549 cells cultured in F12 medium and treated with 10 or 50 nM torin1 for 24 h. HSP90 was used as loading control. (**C**) Viability (luminescence) of F12-cultured A549 cells treated with 10 or 50 nM torin1 for 24 h. (**D**) BSO dose response curves for A549 cells cultured in F12 medium and treated with 10 nM torin1 or control for 72 h. The data were normalized against the mean of the untreated samples for each condition. **(E)** Schematic of the ISR pathway. (**F, G**) Western blotting and quantification of GCN2, p-GCN2, eIF2α, p-eIF2α, ATF4, and CHOP in protein extracts of A549 (F) or H838 (G) cells cultured in F12 or F12AA medium and treated with 100 μM BSO for 24 h. HSP90 was used as loading control. **(H)** Western blotting and quantification of p-GCN2 and ATF4 in protein extracts of A549 cells at 0, 6, 9, 12, 24, and 48 h after switching from F12AA medium to a pre-conditioned F12 medium. (**I**) Schematic model showing methionine abundance, estimated methionine abundance, ATF4 expression, and estimated ISR activity, as indicated. Data on methionine abundance were retrieved from Fig. 3E and ATF4 expression from Fig. 4H. Thresholds for mild and robust ISR activation are indicated by arrows. n = 3 replicates for all datapoints, error bars show SEM. ∗∗∗∗P < 0.0001, ∗∗∗P < 0.001, ∗∗P < 0.01, ∗P < 0.05.

mTORC1 is inactivated at low amino acid concentrations [35]. To assess mTORC1 activity, we investigated phosphorylation of the downstream targets ribosomal protein S6 (S6) and eukaryotic translation initiation factor 4E-binding protein 1 (4E-BP1). There was no decrease in phosphorylation of these proteins in A549 cells that were cultured in F12 versus F12AA or RPMI medium, in the presence or absence of BSO (Fig. 4A). However, total levels of 4E-BP1 were higher in F12 compared to F12AA or RPMI-cultured cells.

To fully exclude the role of mTORC1, we treated A549 cells with increasing concentrations of the mTOR inhibitor torin1 and determined levels of phosphorylated S6 and 4E-BP1 (Fig. 4B). Because torin1 decreased cell viability on its own (Fig. 4C), we could not use high concentrations of torin1 in combination with BSO. Nevertheless, at concentrations that clearly abolished phosphorylation of S6, torin1 had no impact on the IC50 value of BSO (Fig. 4D). Thus, inactivation of mTORC1 does not play a role in this scenario.

The GCN2 kinase is activated by auto-phosphorylation in the presence of uncharged tRNA molecules that accumulate when amino acids become sparse [36,37]. This leads to phosphorylation of eukaryotic translation initiation factor 2α (eIF2α) and activation of the integrated stress response (ISR) pathway (Fig. 4E). Phosphorylated eIF2α inhibits CAP-dependent mRNA translation while stimulating CAP-independent translation of selected proteins including activating transcription factor 4 (ATF4) and C/EBP homologous protein (CHOP, DDIT3), thus orchestrating the ISR [37,38].

Phosphorylation of GCN2 and eIF2α was increased in F12 compared to F12AA-cultured A549 and H838 cells, indicating activation of the ISR pathway (Fig. 4F and G). In agreement, the amount of ATF4 and CHOP was dramatically increased in F12 compared to F12AA-cultured cells (Fig. 4F and G). The expression of these proteins was not affected by BSO treatment, showing that ISR-activation is mediated by low amino acid levels and not by oxidative stress. Activation of the ISR pathway can explain why 4E-BP1, which is an established target of ATF4 [35], showed increased expression in F12 compared to F12AA or RPMI-cultured cells.

eIF2α is activated by additional kinases: RNA-like ER kinase (PERK) in response to endoplasmic reticulum stress, protein kinase R (PKR) in response to virus infection, and eIF2α kinase heme-regulated inhibitor (HRI) in response to heme deprivation (Fig. 4E) [37]. PERK and PKR were phosphorylated in A549 and H838 cells and may thus contribute to ISR activity (Fig. S6A and B). However, there was no difference in phosphorylation of the proteins between cells cultured in F12 and F12AA medium, suggesting that they do not trigger activation of the pathway in F12-cultured cells. HRI was not investigated since it is mainly expressed in erythroid cells [39].

To determine how long it takes to activate the ISR pathway after reducing the amino acid concentration in the medium, we measured ATF4 expression in A549 cells at different time points after switching from F12AA to F12 medium. ATF4 was not upregulated until 48 h after the switch (Fig. S6C) suggesting that amino acid consumption by the cells over time is necessary to reach the threshold concentration in the medium that activates the pathway, or that activation is delayed by cell-intrinsic factors. To distinguish between these possibilities, we repeated the experiment using F12 medium that had been preconditioned with A549 cells for 48 h and included phospho-GCN2 in the analysis. Induction of GCN2 phosphorylation and ATF4 expression was evident at 9 h but not fully developed until 48 h after the media change (Fig. 4H). Thus, activation of the pathway is delayed by cell-intrinsic factors, possibly depletion of intracellular amino acids. Based on our data on methionine abundance (Fig. 3E) and ATF4 expression (Fig. 4H), we propose a parsimonious scenario for GCN2 activation of the ISR-pathway (Fig. 4I): Intracellular concentrations of critical amino acids decline after 6 h to reach a lowest point between 24 and 48 h after media exchange. The ISR pathway is mildly activated between 6 and 9 h and robustly activated after 24 h leading to metabolic adaptations that partly restore amino acid concentrations at 48 h after media exchange.

In a previous publication, the RIPK1 inhibitor necrostatin-1 abolished induction of ATF4 expression in response to cystine starvation in triple-negative breast cancer cells [40], raising the possibility that necrostatin-1 rescued BSO-treated F12-cultured cells (see Fig. 2E) by blocking ATF4 expression, and not by inhibiting necroptosis. To exclude this possibility, F12-cultured A549 cells were treated for 24 h with necrostatin-1. The treatment had no effect on ATF4 expression in F12-cultured cells (Fig. S6D). Similar results were obtained with inhibitors of TNFR1, TLR3, or TLR4 that operate upstream of RIPK1 in necroptosis (Fig. S6D).

### 3.5 Increased autophagy in F12-cultured cells does not influence BSO sensitivity

**Fig. 5:**
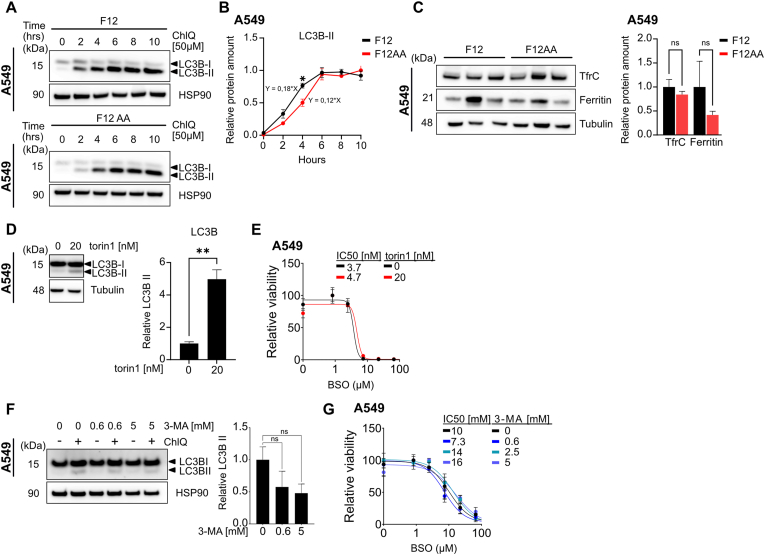
**Increased autophagy in F12-cultured cells does not influence BSO sensitivity**. (**A, B**) Western blotting (A) and quantification (B) of LC3B–I and II expression in protein extracts of A549 cells cultured in F12 or F12AA medium and treated with 50 μM chloroquine (ChlQ) for the indicated time periods. HSP90 was used as loading control. **(C)** Western blotting and quantification of TFRC and Ferritin (heavy chain) in protein extracts of A549 cells cultured in F12 or F12AA medium. Tubulin was used as loading control. **(D)** Western blotting and quantification of LC3B-II in protein extracts of A549 cells cultured in F12 medium and treated with 20 nM torin1 for 24 h. Tubulin was used as loading control. **(E)** Dose response curves for F12-cultured A549 cells treated with BSO in combination with 20 nM torin1 or control for 72 h. (**F**) Western blotting and quantification of LC3B-II expression in protein extracts of A549 cells cultured in F12 medium and treated with 0, 0.6, or 5 mM 3-MA in the presence of 50 μM chloroquine (ChlQ) for 1 h. HSP90 was used as loading control. **(G)** Dose response curves for F12-cultured A549 cells treated with BSO in combination with 0, 0.6, 2.5, or 5 mM 3-MA for 72 h. Dose response curves were normalized against the mean of the untreated samples for each condition. n = 3 replicates for all datapoints, error bars show SEM. ∗∗P < 0.01, ∗P < 0.05.

Activation of the ISR pathway increases autophagy by which cells degrade their own organelles and macromolecules to recycle metabolites [41]. Since autophagy and ferroptosis are causally interconnected [42], autophagy may contribute to greater BSO sensitivity in F12-cultured cells. We used the lipidated isoform of microtubule-associated protein 1A/1B-light chain 3B (LC3B-II) to monitor the amount of autophagosomes, and the lysosome inhibitor chloroquine to estimate autophagic flux. Following addition of chloroquine, levels of LC3B-II increased at a linear rate during 4 h, after which the slope levelled off (Fig. 5A and B). During the linear phase, the accumulation of LC3B-II was faster in F12 (0.18 units/hr [0.14–0.22]) compared to F12AA-cultured cells (0.12 units/hr [0.09–0.14]), indicating a small but significant increase in autophagic flux.

Ferritinophagy, by which cells recycle ferritin, is particularly relevant to ferroptosis because it increases the labile iron pool [43,44]. Although ferritin levels varied between samples, there was no evidence of decreased ferritin levels in F12 compared to F12AA-cultured cells (Fig. 5C), arguing against increased ferritinophagy as a contributing factor. Moreover, there was no difference in transferrin receptor-1 (TFRC) expression between F12 and F12AA-cultured cells (Fig. 5C), excluding another possible cause of altered ferroptosis susceptibility.

To assess the significance of increased autophagic flux, we treated F12-cultured A549 cells with BSO in the presence of chloroquine or bafilomycin, another lysosome inhibitor. Both inhibitors reduced BSO sensitivity suggesting that BSO-lethality may be autophagy-dependent (Fig. S7A). However, prolonged chloroquine treatment (24 h) shut down expression of ATF4 in F12-cultured cells making the data difficult to interpret (Fig. S7B). To clarify the importance of autophagy, we used the mTOR inhibitor torin1 to stimulate macroautophagy. Concentrations of torin1 that increased the amount of autophagosomes, as shown by augmented LC3B-II expression (Fig. 5D), had no effect on BSO sensitivity (Fig. 5E). Moreover, the macroautophagy inhibitor 3-methyladenine (3 MA), which reduced accumulation of LC3B-II in the presence of chloroquine, did not alter the BSO IC50 values (Fig. 5F and G). Overall, we found no compelling evidence that autophagy affects BSO sensitivity in F12-cultured cells. The torin1 data support our previous assessment that mTORC1 is not involved (see Fig. 4B–D).

### 3.6 Activation of the ISR pathway sensitizes lung cancer cells to BSO

**Fig. 6:**
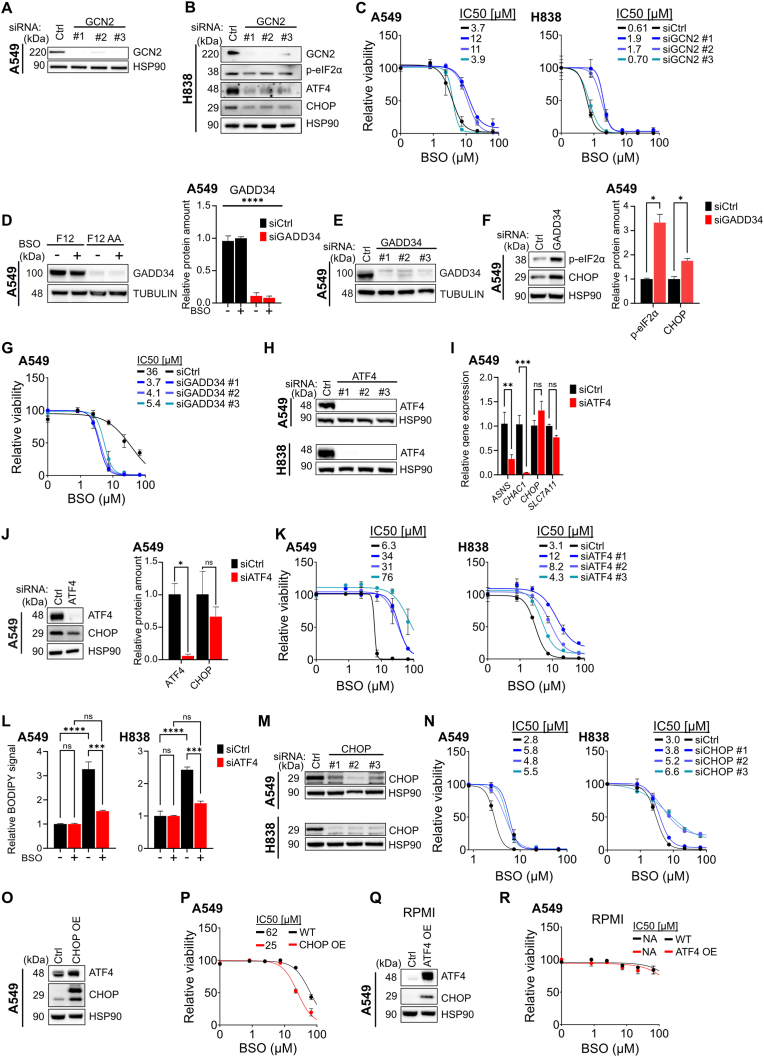
**Activation of the ISR pathway sensitizes lung cancer cells to BSO.** (**A, B**) Western blotting of GCN2 in protein extracts of F12-cultured A549 cells (A) and GCN2, p-eIF2α, ATF4 and CHOP in protein extracts of F12-cultured H838 cells (B) that were transfected with Ctrl siRNA or siRNA targeting GCN2 mRNA. HSP90 was used as loading control. (**C**) BSO dose response curves for A549 (left) and H838 (right) cells cultured in F12 medium for 72 h and transfected with Ctrl siRNA or siRNA targeting GCN2 mRNA. (**D**) Western blotting and quantification of GADD34 in protein extracts of A549 cells that were cultured in F12 or F12AA medium and treated with 100 μM BSO for 24 h. Tubulin was used as loading control. (**E**) Western blotting of GADD34 in protein extracts of F12-cultured A549 cells that were transfected with Ctrl siRNA or siRNA targeting GADD34 mRNA. Tubulin was used as loading control. (**F**) Western blotting and quantification of p-eIF2α and CHOP in protein extracts of F12-cultured A549 cells that were transfected with Ctrl siRNA or siRNA targeting GADD34 mRNA. HSP90 was used as loading control. (**G**) BSO dose response curves for A549 cells cultured in F12 medium for 72 h and transfected with Ctrl siRNA or siRNA targeting GADD34 mRNA. (**H**) Western blotting of ATF4 in protein extracts of F12-cultured A549 (top) or H838 (bottom) cells that were transfected with Ctrl siRNA or siRNA targeting ATF4 mRNA. HSP90 was used as loading control. (**I**) mRNA expression of ASNS, CHAC1, CHOP, and SLC7A11 in F12-cultured A549 cells transfected with Ctrl siRNA or siRNA targeting ATF4 mRNA. GAPDH was used as a reference gene for normalization. (**J**) Western blotting and quantification of ATF4 and CHOP in protein extracts of F12-cultured A549 cells that were transfected with Ctrl siRNA or siRNA targeting ATF4 mRNA. HSP90 was used as loading control. (**K**) BSO dose response curves for A549 (left) and H838 (right) cells cultured in F12 medium for 72 h and transfected with Ctrl siRNA or siRNA targeting ATF4 mRNA. (**L**) Quantification by flow cytometry of oxidized BODIPY-C11 fluorescence in A549 (left) or H838 (right) cells that were cultured in F12 medium and treated with 100 μM BSO or vehicle for 24 h and transfected with Ctrl siRNA or siRNA targeting ATF4 mRNA. (**M**) Western blotting of CHOP in protein extracts of F12-cultured A549 (top) or H838 (bottom) cells that were transfected with Ctrl siRNA or siRNA targeting CHOP mRNA. HSP90 was used as loading control. (**N**) BSO dose response curves for A549 (left) and H838 (right) cells cultured in F12 medium for 72 h and transfected with Ctrl siRNA or siRNA targeting CHOP mRNA. (**O**) Western blotting of ATF4 and CHOP in protein extracts of F12-cultured A549 cells that carried a lentivirus overexpressing CHOP cDNA or control. HSP90 was used as loading control. (**P**) BSO dose response curves for A549 cells that carried lentivirus overexpressing CHOP cDNA or control and were cultured in F12 for 72 h. (**Q**) Western blotting of ATF4 and CHOP in protein extracts of RPMI-cultured A549 cells that carried a lentivirus overexpressing ATF4 cDNA or control. HSP90 was used as loading control. **(R)** BSO dose response curves for A549 cells that carried a lentivirus overexpressing ATF4 cDNA or control and were cultured in RPMI medium for 72 h. Dose response curves were normalized against the mean of the untreated samples for each condition. n = 3 replicates for all datapoints, error bars show SEM. ∗∗∗∗P < 0.0001, ∗∗∗P < 0.001, ∗∗P < 0.01, ∗P < 0.05.

The ISR is mainly an adaptive response that decreases cell stress, but prolonged or excessive activation of the pathway can induce cell death [37]. To investigate whether ISR pathway activation attenuates or induces cell death in the presence of BSO, we inactivated GCN2 in F12-cultured A549 or H838 cells using three independent siRNAs (Fig. 6A and B). Knockdown of GCN2 reduced levels of phosphorylated eIF2α, ATF4, and CHOP, compared to control (Fig. 6B and Fig. S8A), showing that GCN2 is required for ISR pathway activation in F12-cultured cells. Two siRNA markedly reduced cell death in the presence of BSO suggesting that ISR pathway activation is required for BSO-lethality (Fig. 6C). One of the siRNAs did not affect BSO sensitivity, possibly reflecting off-target toxicity.

**Fig. 7:**
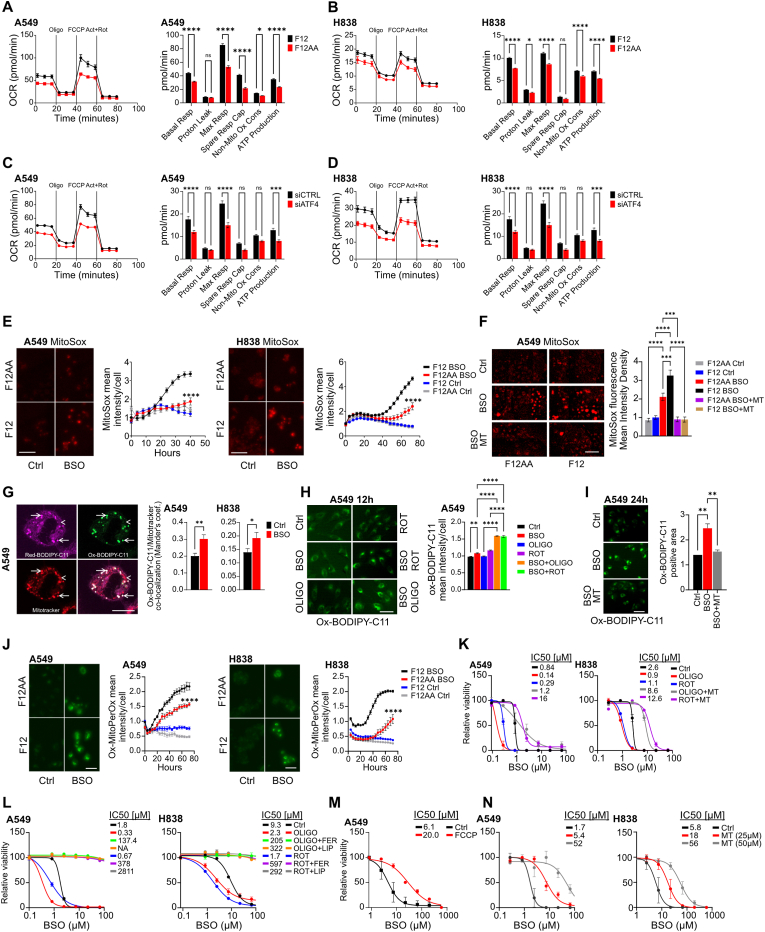
**Increased mitochondrial respiration renders F12-cultured cells BSO-sensitive.** (**A, B**) Oxygen consumption rate (left) in F12 or F12AA-cultured A549 (A) or H838 (B) cells treated with 0.5 μM oligomycin (Oligo), 1 μM FCCP, and 0.5 μM rotenone (Rot), as indicated. Graphs (right) showing parameter data extracted from the oxygen consumption rate and extracellular acidification rate (n = 14 in A549 cells; n = 10 in F12 and 4 in F12AA-cultured H838 cells). (**C**, **D**) Oxygen consumption rate (left) in F12-cultured A549 (C) or H838 (D) cells transfected with control siRNA or siRNA targeting ATF4 mRNA and assayed as in (A, B). Graphs (right) show extracted parameters (n = 15 for A549; n = 21 for ATF4 siRNA and n = 13 for control siRNA in H838). (**E**) MitoSox fluorescence images of A549 (left) and H838 (right) cells cultured in F12 or F12AA medium. Cells were treated with 30 μM BSO for 40 h (A549) or 48 h (H838). Graphs show IncuCyte-based fluorescence quantification over time. Scale bar, 100 μm. (**F**) MitoSox fluorescence images of A549 cells cultured in F12 or F12AA medium and treated for 24h with 50 μM BSO, 50 μM BSO + 20 μM mito-TEMPO (MT), or control. MitoSox was added during the last 90 min of treatment; cells were then washed and quantified by live imaging. The graph shows IncuCyte-based fluorescence quantification. Scale bar, 100 μm; n = 8–10. (**G**) Confocal microscopy images of F12-cultured A549 cells treated with 100 μM BSO for 24h showing reduced (pink) and oxidized (green) BODIPY-C11 in combination with mitotracker deep red (red). Corresponding graphs show pixel-wise colocalization (Mander's coefficient) of oxidized BODIPY-C11 and mitotracker deep red in F12-cultured A549 (left) or H838 (right) cells treated with 100 μM BSO for 24 h or controls. n = 6–9 visual fields in A549 cells and 48 visual fields in H838 cells. Scale bar, 10 μm. (**H**) Oxidized BODIPY-C11 fluorescence images of A549 cells cultured in F12 medium and treated for 12 h with 100 μM BSO, 100 nM rotenone, 100 nM oligomycin, or combinations of BSO + rotenone or BSO + oligomycin, and controls. Graph shows IncuCyte-based fluorescence quantification. Scale bar, 50 μm. (**I**) Oxidized BODIPY-C11 fluorescence images of A549 cells cultured in F12 medium and treated for 24 h with 100 μM BSO, BSO + 20 μM mito-TEMPO (MT), or control. Graph shows IncuCyte-based fluorescence quantification. Scale bar, 100 μm. (**J**) Oxidized MitoPerOx fluorescence images of A549 (left) and H838 (right) cells cultured in F12 or F12AA medium. Cells were treated with 30 μM BSO or control for 48 h. Graphs show IncuCyte-based fluorescence quantification over time. Scale bar, 100 μm. (**K)** Dose response curves for F12-cultured A549 or H838 cells treated with BSO in combination with 100 nM oligomycin, 100 nM rotenone, 20 μM mito-TEMPO + rotenone, or mito-TEMPO + oligomycin, or control for 72 h. (**L**) Dose response curves for F12-cultured A549 or H838 cells treated with BSO in combination with 100 nM oligomycin, 100 nM rotenone, 5 μM ferrostatin-1 (FER) + rotenone, 5 μM liproxstatin-1 (LIP) + rotenone, ferrostatin-1 + oligomycin, or liproxstatin-1 + oligomycin, or control for 72 h. (**M**) Dose response curves of F12-cultured A549 cells treated with BSO in combination with 0.5 μM FCCP, or control for 72 h. (**N**) Dose response curves for F12-cultured A549 or H838 cells treated with BSO in combination with 25 or 50 μM mito-TEMPO, or control for 72 h. Dose response curves were normalized against the mean of the untreated samples for each condition. n = 3 replicates for all datapoints unless otherwise indicated. Error bars show SEM. ∗∗∗∗P < 0.0001, ∗∗∗P < 0.001, ∗∗P < 0.01, ∗P < 0.05.

To further assess the impact of ISR-pathway activation, we investigated DNA damage-inducible protein 34 (GADD34, PPP1R15A), a negative feedback regulator of the ISR pathway that dephosphorylates p-eIF2α (see Fig. 4E) [45]. Expression of GADD34 was markedly higher in F12 compared to F12-cultured cells (Fig. 6D and Fig. S8B) and knockdown of GADD34 in F12-cultured cells increased phosphorylation of eIF2α, expression of the downstream effector CHOP, and sensitivity towards BSO (Fig. 6E–G and Fig. S8C), in line with the GCN2 data.

ATF4 orchestrates a transcriptional response downstream of the ISR pathway. To assess the role of ATF4-driven transcription, we knocked down ATF4 (Fig. 6H) and investigated mRNA expression of known ATF4 targets [[46], [47], [48], [49]]. Knockdown of ATF4 reduced expression of ASNS and CHAC1, but had no effect on CHOP or SLC7A11 (Fig. 6I), suggesting that ATF4 regulates a subset of known targets in F12-cultured cells. Lack of CHOP regulation was confirmed by western blotting (Fig. 6J). Knockdown of ATF4 had two effects on cell viability, one in the absence of BSO and one in the presence. In the absence of BSO, knockdown of ATF4 reduced the number of cells, compared to controls, suggesting that ATF4 adapts cells to the low amino acid content in F12 medium (Fig. S8D). At the same time, ATF4-deficient cells were markedly resistant to BSO (Fig. 6K) showing that a transcriptional response downstream of ATF4 is required for BSO-toxicity. In accord, knockdown of ATF4 prevented lipid peroxidation in the presence of BSO (Fig. 6L).

By degrading glutathione, CHAC1 (glutathione-specific γ-glutamylcyclotransferase 1) increases ferroptosis susceptibility in other models [40,[49], [50], [51]], suggesting that ATF4-driven CHAC1 expression might render F12-cultured cells BSO sensitive. However, protein levels of CHAC1 were equal in F12 and F12AA-cultured cells (Fig. S8E) and knockdown of CHAC1 mRNA with three independent siRNAs had no impact on BSO sensitivity in F12-cultured cells (Fig. S8F and G). Thus, CHAC1 is not playing an important role in this model. In agreement, glutathione concentrations were equal in F12 compared to F12AA-cultured cells (Fig. 3I).

The transcription factor CHOP can induce cell death in response to excessive activation of the ISR pathway [40]. In the absence of BSO, transfection with siRNA against CHOP reduced the number of H838 cells but had no effect on A549 cells, compared to control (Fig. 6M, Fig. S8H). In the presence of BSO, inactivation of CHOP conferred partial BSO resistance in both cell lines (Fig. 6N). The data suggest that CHOP contributes to BSO sensitivity but not to the same extent as ATF4. However, the inactivation of CHOP was incomplete making the comparison unreliable. To further assess the importance of CHOP, we overexpressed lentiviral CHOP in F12-cultured A549 cells and established BSO dose response curves. CHOP overexpression had a small but significant effect on BSO sensitivity (Fig. 6O and P), confirming that CHOP contributes to BSO sensitivity.

To investigate whether expression of ATF4 and CHOP is sufficient to induce BSO sensitivity in RPMI-cultured cells, we infected A549 cells with lentivirus expressing ATF4. The transduced cells overexpressed ATF4 and CHOP (Fig. 6Q), consistent with CHOP being an established downstream target of ATF4 in many instances [52]. Overexpression of ATF4 and CHOP had no impact on BSO sensitivity in RPMI-cultured cells (Fig. 6R), indicating that amino acid deprivation has additional effects necessary for increasing ferroptosis susceptibility.

We conclude that ATF4 and CHOP orchestrate a transcriptional response downstream of the ISR pathway that help cells to cope with the amino acid sparsity in F12 medium, at the expense of increased susceptibility to lipid peroxidation and ferroptosis-inducing agents such as BSO.

### 3.7 Increased mitochondrial respiration renders F12-cultured cells BSO-sensitive

Cysteine-deprivation-induced ferroptosis requires mitochondrial respiration [53,54]. To examine whether mitochondrial function contributes to BSO sensitivity, we measured oxygen consumption and extracellular acidification in A549 and H838 cells cultured in F12 or F12AA medium. Because the assay measures changes in dissolved oxygen and protons, which are highly sensitive to the buffering capacity of the medium, it must be performed in a standardized medium. We therefore replaced F12 and F12AA with the recommended assay medium (Seahorse XF RPMI Medium) during the analysis.

F12-cultured cells showed increased basal and maximal oxygen consumption compared to cells grown in F12AA medium (Fig. 7A and B). ATP production was also elevated in F12-cultured cells (Fig. 7A and B). Moreover, F12-culture increased the extracellular acidification rate compared to F12AA-culture (Fig. S9A and B), suggesting that culture in F12 medium shifted the cells to a more energetic state rather than causing a shift from anaerobic to aerobic respiration. Transfection with siRNA against ATF4 markedly decreased basal and maximal oxygen consumption in F12-cultured cells compared to controls, and reduced ATP-production and extracellular acidification (Fig. 7C, D and Fig. S9C and D). Thus, increased mitochondrial respiration in F12-cultured cells requires ATF4 expression.

Given that mitochondrial respiration produces superoxide at multiple sites, which is rapidly converted to membrane-permeable hydrogen peroxide (H_2_O_2_) capable of inducing lipid peroxidation [[55], [56], [57]], we next investigated whether culture in F12 medium increases mitochondrial superoxide levels using MitoSox, a mitochondria-targeted superoxide indicator. BSO treatment markedly increased MitoSox staining in cells cultured in F12 compared with F12AA medium (Fig. 7E). MitoSox induction was further accelerated by the ATP synthase inhibitor oligomycin (Fig. S9E), which enhances superoxide leakage from the electron transport chain [58]. Notably, the MitoSox signal was completely eliminated by mito-TEMPO, a mitochondria-targeted superoxide scavenger (Fig. 7F and Fig. S9E).

We next examined the spatial relationship between mitochondrial ROS and lipid peroxidation. Using ratiometric confocal microscopy, we found that F12-cultured cells accumulated oxidized BODIPY-C11 within vesicles that overlapped with a subset of mitochondria, as evidenced by colocalization with mitotracker deep red (Fig. 7G and Fig. S9F). The overlap between oxidized BODIPY-C11 and mitochondria was enhanced by BSO treatment, as shown with pixel-wise colocalization analysis (Fig. 7G). Note that pixel-wise colocalization analysis was performed over entire images and not for regions of interest. Oxidation of BODIPY-C11 in BSO-treated cells was accelerated by oligomycin or the complex I inhibitor rotenone (Fig. 7H), which causes superoxide leakage from the electron transport chain [57,59], and abolished by mito-TEMPO (Fig. 7I). To further assess mitochondrial lipid peroxidation, we used MitoPerOx, a mitochondria-targeted ratiometric lipid peroxide probe. High-resolution confocal microscopy confirmed mitochondrial localization of the probe (Fig. S9G). Live-imaging fluorescence quantification revealed greater MitoPerOx oxidation in BSO-treated cells compared with controls, with higher oxidation in BSO-treated cells cultured in F12 medium than in F12AA medium (Fig. 7J).

To determine whether elevated mitochondrial ROS contributes causally to ferroptosis susceptibility, we treated cells with oligomycin or rotenone. Both agents markedly increased BSO sensitivity in F12-cultured cells, and this effect was abolished by mito-TEMPO, indicating that mitochondrial ROS is a rate-limiting factor in this context (Fig. 7K). The sensitizing effects of oligomycin and rotenone were fully reversed by the lipid peroxide scavengers ferrostatin-1 and liproxstatin-1 (Fig. 7L), and partially attenuated by the iron chelator deferoxamine (Fig. S9H), supporting a role for iron-dependent lipid peroxidation. In agreement, treatment with the mitochondrial uncoupler FCCP, which reduces ROS production from the electron transport chain [60], restored viability in BSO-treated A549 cells but had no effect in H838 cells (Fig. 7M and Fig. S9I). Finally, mito-TEMPO rescued cell viability in a dose-dependent manner following BSO treatment (Fig. 7N), confirming that scavenging mitochondrial superoxide mitigates BSO-induced ferroptosis sensitivity.

We conclude that culture in F12 medium increases mitochondrial respiration in an ATF4-dependent manner. This increase in respiration promotes ROS production from mitochondria in glutathione-depleted cells, thus increasing the sensitivity to ferroptosis-inducing agents.

## 4 Discussion

**Fig. 8:**
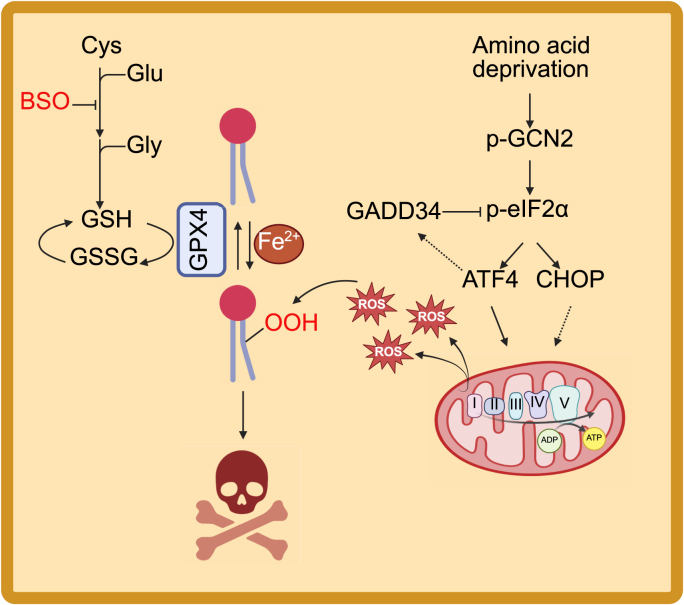
Schematic showing how amino acid restriction sensitizes lung cancer cells to ferroptosis-inducing agents by GCN2-dependent activation of the ISR.

Glutathione is the most abundant intracellular antioxidant, yet glutathione biosynthesis blockade has little impact on the viability of human cultured cancer cells [[12], [13], [14]]. In the present study, we found that levels of extracellular amino acids markedly impact the susceptibility to glutathione depletion in human lung cancer cells. A reduction in the amount of extracellular amino acids activates GCN2 and the ISR pathway, thus sensitizing cancer cells to iron-dependent lipid peroxidation and cell death in the presence of BSO (Fig. 8).

Ferroptosis susceptibility has been previously linked to perturbations limiting glutathione biosynthesis, including cystine or cysteine depletion [[61], [62], [63]], pharmacological blockade of cystine uptake by the system-xc inhibitor erastin [10,64], or methionine depletion that blocks generation of cysteine through the transsulfuration pathway [19,65,66]. The mechanism we describe is uncoupled from cysteine metabolism and glutathione biosynthesis. The concentration of cysteine was equal in F12 and F12AA media, there was no significant difference in intracellular concentrations of cysteine, glycine or glutamate between F12 and F12AA-cultured cells, and there was no difference in glutathione concentration between BSO-treated F12 and F12AA-cultured cells. Instead, ferroptosis susceptibility was linked to glutathione-dependency: At lower concentrations of amino acids, cells become dependent on glutathione to prevent accumulation of lipid peroxides.

Induction of ferroptosis through cysteine or cystine depletion, erastin treatment, or genetic inactivation of system-xc is known to concurrently activate the ISR pathway, but the significance of this activation has not been thoroughly investigated [40,64,[67], [68], [69], [70]]. Our findings shed new light on these observations and on the acknowledged mystery of why depletion of cysteine (or cystine) leads to worse ferroptosis compared to glutathione synthesis blockade alone [[12], [13], [14],^63^,[69], [70], [71], [72], [73]]: Besides limiting glutathione biosynthesis, cysteine depletion activates the ISR pathway, thus inducing glutathione-dependency. Notably, BSO has been reported to activate the ISR pathway in certain contexts [74]; however, in our experiments, ISR pathway activity was unaffected by BSO treatment.

Four eIF2a kinases – GCN2, PERK, PKR or HRI – mediate the activation of the ISR Pathway [37]. Several lines of evidence indicate that GCN2 is the activator in F12-cultured cells: F12-culture induces GCN2 autophosphorylation, knockdown of GCN2 attenuates the expression of ISR markers, and GCN2 knockdown rescues F12-cultured cells in the presence of BSO. PERK and PKR likely feed into the phosphorylation of eIF2α but their activity was not altered by F12-culture. GCN2 is activated by accumulation of uncharged tRNA molecules or by ribosomal stalling or collision [[75], [76], [77]]. We favour a scenario in which GCN2 activation is caused by intracellular depletion of several amino acids – specially methionine, serine, leucine, and isoleucine whose levels inversely correlated with GCN2 phosphorylation – and accumulation of the cognate uncharged tRNAs. The kinetics of amino acid depletion and p-GCN2/ATF4 induction upon transfer from RPMI to F12 medium indicates multiple activation thresholds of the ISR (Fig. 4I), supporting the idea that ISR outcomes diversify – from protective to lethal – with stress intensity and duration [37]. Notably, ISR activation in F12-cultured cells was protective, except under glutathione depletion.

The transcription factors ATF4 and CHOP are translated from internal ribosomal entry sequences in response to eIF2α phosphorylation [35,38]. CHOP, which is a transcriptional target of ATF4 and often positioned downstream of ATF4 in the ISR pathway [52], was regulated independently of ATF4 in F12-cultured cells. Knockdown of ATF4 or CHOP reduced BSO sensitivity, demonstrating that both proteins are required to achieve maximal ferroptosis sensitivity. Since ATF4 and CHOP form heterodimers that co-regulate many genes [78,79], the proteins may act in concert. However, inactivation of ATF4 had a greater impact on BSO sensitivity than CHOP inactivation, suggesting that ATF4 plays a more important role. Overexpression of ATF4 and CHOP was not sufficient to increase BSO sensitivity in RPMI-cultured cells, demonstrating that F12-culture triggers additional events.

We found that ATF4 expression enhances mitochondrial respiration in F12-cultured cells. Based on several lines of evidence, we propose that ferroptosis susceptibility is caused by increased ROS leakage from mitochondria: (1) BSO treatment elevates mitochondrial superoxide levels more strongly in F12 than in F12AA-cultured cells. (2) In the presence of BSO, oxidized BODIPY-C11 colocalizes with mitochondria, thus demonstrating spatial correlation between lipid peroxidation and mitochondria. This finding is further supported by MitoPerOx, a mitochondria-targeted lipid peroxide probe. (3) Rotenone or oligomycin, which promote ROS leakage from the electron transport chain, enhanced iron- and lipid peroxidation-dependent cell death in the presence of BSO, while FCCP treatment (which reduces ROS leakage) made A549 cells less BSO-sensitive. (4) mito-TEMPO, a mitochondria-targeted superoxide scavenger, rescued the viability of BSO-treated cells. In agreement, ROS generation from mitochondria is an established driver of ferroptosis susceptibility [11,53,80,81].

ATF4, a central regulator of the mitochondrial ISR, coordinates nuclear-mitochondrial antegrade signaling to adapt metabolic function [82]. Accumulating evidence links ATF4 or CHOP expression with enhanced mitochondrial respiration [83,84]. For example, under nutrient or ER stress, activation of the PERK/p-eIF2α/ATF4 pathway promotes SCAF1-dependent assembly of respiratory supercomplexes, increasing oxidative phosphorylation [85]. In lung macrophages from individuals with asbestosis, ATF4 directly binds the PGC-1α promoter, driving mitochondrial biogenesis and respiration [86]. Conversely, ATF4 or CHOP activation can also suppress mitochondrial respiration [87,88]. Thus, defining the contextual factors that determine ATF4 and CHOP effects on mitochondrial function remains an important direction for future investigation.

BSO is a classic ferroptosis-inducing agent but can also lead to other forms of cell death. Necroptosis is triggered by stimulation of TNFR1 or TLRs and mediated by RIPK1 [31], inhibitors of which rescued BSO-treated cells. Hence, it is possible that necroptosis is part of the killing mechanism. Oxidized phospholipids and their degradation products malondialdehyde or 4-hydroxynonenal serve as damage associated molecular patterns (DAMPs) for the regulation of innate immunity by pattern recognition receptors such as TLR3 and TLR4 [89]. Moreover, oxidized 1-palmitoyl-2-arachidonoyl-phosphatidylcholine induces lung injury and cytokine production in mice, in a TLR4-dependent manner [90]. Thus, oxidized phospholipids may directly or indirectly stimulate necroptosis-inducing receptors. However, the distinction between ferroptosis and necroptosis is currently vague and should not be overinterpreted [11].

ATF4 can influence ferroptosis susceptibility by preserving cysteine. On one hand, ATF4 stimulates conversion of serine to cysteine through the transsulfuration pathway and cystine import through system-xc. These actions promote glutathione production and protects against ferroptosis [[61], [62], [63], [64], [65], [66], [67]]. On the other hand, ATF4 promotes cysteine salvage from glutathione by upregulating CHAC1*,* a glutathione-degrading enzyme that increases susceptibility to ferroptosis [40,[49], [50], [51]]. However, in our experiments, CHAC1 is not rate-limiting for ferroptosis susceptibility. Prolonged methionine deprivation blocks translation of CHAC1 mRNA in human HT-1080 cells and in mouse Hepa1-6, MC38, and PanO2 cells, thereby uncoupling protein abundance from mRNA regulation [51,65]. It is possible that methionine depletion in F12-cultured cells produces a similar effect.

mTORC1, which regulates ferroptosis susceptibility in other contexts, did not affect BSO sensitivity in F12-cultured cells. Macroautophagy and ferritinophagy also appeared dispensable. However, chloroquine or bafilomycin treatment rescued cell viability in the presence of BSO, suggesting that lysosomal activity contributes in other ways. Chloroquine suppressed ATF4 expression implying that chloroquine primarily dampens ISR signalling. Moreover, as chloroquine inhibits TLR3/4 signaling required for BSO-induced death [[91], [92], [93], [94]], its protective effects may involve anti-inflammatory mechanisms.

Nutrient stress is common in tumors due to poor vascularization. Efforts to restore homeostasis by inducing the ISR pathway may collaterally sensitize tumor cells to lipid peroxidation making the cells susceptible to ferroptosis, as shown here. Insufficient amino acid supplies may therefore contribute to the oxidative stress weakness that is evident in many tumors [6,7,[95], [96], [97]]. In agreement, serine and glycine restricted diets hampered formation of lymphomas and intestinal tumors in mice, in a ROS-dependent manner [98,99]. Similarly, cysteine and methionine restricted diets potentiated the effect of ferroptosis-inducing agents on glioma and skin cancer development in mice [51,65]. Whether a protein restricted diet potentiates the anti-cancer effect of BSO remains to be tested.

Cell density strongly influences BSO sensitivity in our model. This is expected because Hippo signalling, which is activated to varying degrees by cell to cell contacts depending on density, is a key regulator of ferroptosis susceptibility [100]. To minimize variability, strict control of cell numbers was maintained. Despite this, BSO IC50 values showed some variation over time for reasons that remain unclear. Importantly, the fold change in IC50 values between F12 and F12AA-cultured cells was highly reproducible and remained consistent across all experiments.

Whether amino acid restriction affects ferroptosis susceptibility of non-cancerous cells remains to be tested. Of note, the BSO-sensitizing effect of amino acid restriction was evolutionarily conserved in mouse lung cancer cells (KP cells) and present in SKNBE-2 neuroblastoma cells, indicating that the mechanism might be widespread.

In summary, we show that a mild reduction in levels of extracellular amino acids sensitizes lung cancer cells to ferroptosis-inducing agents by upregulating the ISR pathway. The finding opens new opportunities for dietary amino acid restriction to modify ferroptosis susceptibility in cancer patients.

## CRediT authorship contribution statement

**Viktor Antonsson Garellick:** Conceptualization, Investigation, Visualization, Writing – original draft, Writing – review & editing. **Nadia Gul:** Conceptualization, Investigation, Supervision. **Parvin Horrieh:** Investigation. **Dyar Mustafa:** Investigation. **Angana A.H. Patel:** Investigation. **Martin Dankis:** Investigation. **Samantha W. Alvarez:** Investigation. **Johanna Berndtson:** Investigation. **Saeed Mahdavi:** Investigation. **Maria Schwarz:** Investigation. **Andreas Persson:** Investigation. **Fikret Zahirovic:** Investigation. **Clotilde Wiel:** Conceptualization, Funding acquisition, Investigation, Supervision, Writing – review & editing. **Volkan I. Sayin:** Conceptualization, Funding acquisition, Supervision, Writing – review & editing. **Per Lindahl:** Conceptualization, Funding acquisition, Investigation, Project administration, Supervision, Visualization, Writing – original draft, Writing – review & editing.

## Declaration of competing interest

The authors declare the following financial interests/personal relationships which may be considered as potential competing interests: Nadia Gul reports financial support was provided by Asta-Zeneca. If there are other authors, they declare that they have no known competing financial interests or personal relationships that could have appeared to influence the work reported in this paper.

## Data Availability

No data was used for the research described in the article.
